# A school-based epidemiological field survey: difficulties in collecting psychiatric outcome data in a middle-income country

**DOI:** 10.1186/s12888-017-1436-6

**Published:** 2017-07-28

**Authors:** T. M. Fidalgo, Z. M. Sanchez, M. Ribeiro, S. R. Healy, S.C. Caetano, S. S. Martins

**Affiliations:** 1 Department of Psychiatry, Universidade Federal de São Paulo, Avenida Prof. Ascendino Reis 763, São Paulo, SP Brazil; 2 Department of Preventive Medicine, Universidade Federal de São Paulo., São Paulo, Brazil; 3 Epidemiology Department, Mailman School of Public Health Columbia University, New york, USA

**Keywords:** Adolescent psychiatry, Epidemiology, Methodology, Violence

## Abstract

**Background:**

Low- and middle-income countries (LMICs) face a lack of epidemiological data. The development of high-quality surveys is a key research priority in countries such as Brazil. Our aim is to discuss the difficulties in conducting a longitudinal epidemiological survey in a pilot study of a school-based sample in São Paulo.

**Methods:**

Data came from a cohort of school-attending adolescents in two neighborhoods with different levels of urbanicity in São Paulo. Students born in 2002 and in the 7th grade during 2014 were recruited from nine public schools. Adolescents and caregivers were interviewed separately at baseline and at one year follow-up, using several instruments, including the Schedule for Affective Disorders and Schizophrenia for School-Age Children/Present and Lifetime Version (K-SADS-PL).

**Results:**

Achieving unbiased sampling, keeping an updated register of participants’ contact information, using a full clinical interview without an algorithm for its scoring, and maintaining a highly-trained research team were among the difficulties faced.

**Conclusion:**

Working closely with community leaders, organizing group efforts to perform interviews, using a short, easy to understand instrument and providing some reward for participants were identified as alternatives to dealing with these difficulties, useful not only in Brazil, but also in other LMICs.

## Background

To reduce the burden of illness in a cost-effective manner, public policies targeting health issues should be based on scientific evidence [1, 2]. Epidemiological studies are powerful tools that provide a precise assessment of the health status of a population, and, thus, are of great value for policymakers, informing decisions of where and how to invest resources. Although such investigations have been widely conducted in high-income countries, low- and middle-income countries (LMICs) still face a serious lack of accurate epidemiological data. The field of child and adolescent psychiatry (CAP) is no exception; and there are still fewer studies assessing the potential impact of social inequalities and exposure to traumatic experiences on psychiatric outcomes at the population level [3].

Therefore, the development of consistent high-quality epidemiological surveys is one of the key research priorities in LMICs [4, 5]. In Brazil - a middle income country [6] - most studies conducted to date have several serious methodological issues which work to undermine their epidemiological rigor. For instance, in most studies diagnostic ascertainment (if considered at all) is based solely on the primary caregiver interview. More commonly though, there is widespread use of screening instruments, such as the Child Behavior Checklist (CBCL) and the Strengths and Difficulties Questionnaire (SDQ), instead of the use of diagnostic instruments [7–12]. To date, only one Brazilian study [13] used a diagnostic instrument, namely the Development and Well-Being Assessment (DAWBA). Importantly in our view, this study did not evaluate the impact of social conditions and violent experiences on mental health outcomes. Finally, and perhaps most importantly, all of these studies presented only cross-sectional data [7–13] which limits researchers’ ability to accurately determine causality.

Several explanations for the low number of epidemiological studies in psychiatry, particularly in CAP, in LMICs exist, including difficulties in recruitment and follow-up of subjects (often due deficient telephone, internet, and postal services), issues in obtaining valid and reliable data, mainly because of lack of trained personnel, as well as difficulties in standardizing procedures and systems for data imputation [14]. Therefore, the aim of this paper is to discuss the difficulties and feasibility of conducting a CAP longitudinal epidemiological survey in a pilot study of a school-based sample in the city of São Paulo, Brazil.

## Methods

### Study design and sample selection

The data derive from a cohort of school-attending adolescents in two different neighborhoods in São Paulo city. One neighborhood has low exposure to urban violence and scores high on the Human Development Index (HDI^1^), while the other experiences high exposure to urban violence and scores low on the HDI. In total, nine public schools from these neighborhoods were selected for study inclusion. The schools located in the most socially vulnerable regions of each neighborhood were selected.

All students born in 2002 and enrolled in the 7th grade during 2014 were eligible for recruitment and received a letter, explaining the study’s research goals and procedures and providing the research team’s contact information, to bring home to their caregiver. In addition, school-meetings for caregivers were scheduled in four schools. Attendance at these school meetings by parents/caregivers was quite low, however. In the other five schools, the principals did not agree to schedule such meetings between the research team and parents. Subsequently, the chief field supervisor (CFS) telephoned all the caregivers to invite them to participate and to assuage any concerns regarding their participation in the study.

After the State Education Secretariat’s research approval, principals provided caregivers’ phone numbers, available via school records; however, many numbers were out-of-date. We requested that principals attempt to update this contact information but most of the principals were not able to do so because of their already substantial workload. Instead, the CFS visited the schools several times to ask the students directly for their caregivers’ current phone number. Even with these efforts, which took a total of 40 h of fieldwork, of the 416 registered students, only 210 provided an up-to-date landline or cell phone number. Despite the lack of reliable telephone numbers for participants, other modes of contact were even less feasible. Electronic communication (i.e. email) did not seem to be prudent as only 50% of Brazilian households have a computer or an equivalent device (notebook or tablet) and only 50% have access to an internet connection (TIC Domicílios, [15]). In addition, only 60% of Brazilian people have ever used a computer or have ever used an internet connection. These rates are lower for those from low SES (only 28% of Brazilians from low SES have ever used an internet connection (TIC Domicílios, [15]). Finally, only 64% of Brazilians use the internet to access email accounts (38% of those from low SES - TIC Domicílios, [15]).

Recruitment phone calls were made by the CFS. On average, three phone calls were made per hour, for a total of approximately 60 h of fieldwork. Among the 210 students with adequate contact information, 180 agreed to participate (85% acceptance rate). Baseline interviews were conducted from August 2014 to December 2014. For those who attended the baseline interview, at least five phone numbers (of relatives or other contacts) were obtained to facilitate contact at the time of follow-up.

Follow-up evaluations were conducted a year later and procedures were identical to the baseline evaluations. Loss-to-follow-up did not appear to be influenced by diagnostic status, as there were no differences in the K-SADS diagnostic scores between the adolescents who completed the follow-up and those who did not. The CFS called all phone numbers recorded at baseline. For those participants whose numbers were inactive, the CFS visited their schools again and asked the students directly for an active phone number. Approximately 50 h were spent on these new visits. School principals were also encouraged to engage caregivers involved in the research project. Follow-up interviews started in August 2015 and finished in July 2016. From December 2015 to January 2016 no interviews were conducted due to summer vacation.

### Data collection and instruments

The study was explained to both the adolescent and his/her caregiver. The adolescent had to sign the assent form, which expresses willingness to participate in research by persons who are too young to give informed consent (i.e. not legally responsible for themselves), but who are old enough to understand the research aims and activities expected of them as subjects, as well as the risks and benefits of the study. In addition, the caregiver had to sign a consent form, the voluntary agreement of an individual, or his or her authorized representative, who has the legal capacity to give consent, and who exercises free power of choice, without undue inducement or any other form of constraint or coercion, to participate in research [16]. The consent procedure took 30 min on average.

The adolescent and his/her caregivers were interviewed separately by different interviewers. Both face-to-face interviews were conducted by a trained team of child and adolescent psychiatrists and psychologists using LUMIA 635 cell phones, which contained a digital application in which to input questionnaire responses. The caregiver survey comprised demographic information, social capital and social support questions, neighborhood characteristics questions [17], mood, anxiety and substance use screening items from The World Mental Health Composite International Diagnostic Interview (WMH-CIDI), screening and diagnostic modules for Attention-deficit/hyperactivity disorder (ADHD), Oppositional Defiant Disorder (ODD), Conduct Disorder (CD), Major depressive disorder (MDD), Generalized Anxiety Disorder (GAD), Post-traumatic stress disorder (PTSD) and Substance use disorders (SUD) from the Schedule for Affective Disorders and Schizophrenia for School-Age Children/Present and Lifetime Version (K-SADS-PL) and finally information concerning risk perception of substance use. The adolescent survey comprised demographic information, the same screening and diagnostic modules from K-SADS-PL outlined above, information concerning risk perception of substance use and questions regarding pubertal development and sexual behavior.

The K-SADS-PL is a semi-structured psychiatric interview that ascertains both lifetime and current diagnostic status based on the Diagnostic and Statistical Manual of Psychiatric Disorders - Fourth Edition (DSM-IV) criteria. It has been used worldwide as the gold-standard interview for the diagnosis of mental disorders in children aged 6–18 years and has been translated into Brazilian Portuguese [18]. It includes an introductory interview, a screening interview, and diagnostic supplements. It is designed to be used by trained clinicians. Training includes one day focusing on the theory and application of the instrument and two days of practical training using videotaped interviews and shadowing of a senior interviewer. Each interviewer had to watch at least seven different videotaped interviews, each one with a child or adolescent with and without an Axis I DSM-IV diagnosis (bipolar disorder, ADHD, depression, etc.). Interviewers had to achieve at least 80% reliability to be included in the field team.

After interviews are completed, data must be reviewed by a K-SADS-PL-trained and licensed child and adolescent psychiatrist. No standardized scoring method is available, rather the final diagnosis depends on clinical judgment. Reaching the result is a complex manual process, which takes at least one hour per interview and which cannot yet be properly substituted by a computerized algorithm [19].

The entire interview ranged from one to two hours, depending on the number of K-SADS-PL supplements that needed to be completed. For each positive screening section, the corresponding diagnostic supplement was completed.

### Data analysis

Every week, the CFS prepared journals reporting the progress of the fieldwork, including phone calls, visits to the schools and interviews with adolescents and their parents. Besides the CFS’s personal observations, these journals included the impressions of the interviewers and of the schools’ principals. We describe the results qualitatively, based on these field journals.

### Ethical aspects

The protocol was reviewed and approved by the Columbia University Institutional Review Board (IRB- AAM4702) and by the Universidade Federal de São Paulo Research Ethics Committee (Protocol#451.565 of 11/08/2013). The research goals were explained to both the youth and to his/her caregiver. Assent was obtained verbally from the youth and informed consent was obtained in writing from the caregivers (parents or legal guardians), on behalf of the youth.

## Results

Several difficulties were faced in the implementation of the study protocol. These difficulties were largely related to either fieldwork or to instrument characteristics. Thorough information on the problems encountered is provided below.

### Sampling

Among the 416 students on the lists provided by administrators, there were four students who had been transferred but whose new school we could not identify. Moreover, some classes’ records contained names of students who had never actually studied in those schools. Inaccurate lists of enrolled students were a major source of concern as the randomized sampling strategy was based on these outdated lists (around one third of the lists were outdated), which included the names of the transferred, quitter or non-existent students. In larger schools, with more than 100 students, this may not have been a problem, as new draws and replacements could occur. However, in small schools (three out of the nine included), which had less than 100 students, sampling probabilities could have been affected, as there were fewer possibilities for replacement.

Another significant issue was that most schools did not keep an updated database of the registered students’ contact information. Even with the procedure previously described, several numbers could not be updated. During the baseline interview, five additional phone contacts (of relatives or neighbors) were requested, in order to make it possible to reach students and their caregivers during follow-up. However, most of the provided numbers were no longer active at the follow-up. In some cases, none of the provided numbers were active at the time of follow-up.

In thinking about our initial low participation in the recruitment phase, it is important to consider that at least part of our failure in getting accurate phone numbers might be due to passive refusal. The necessity of weekend scheduling to accommodate the majority of parents’ working schedules may have made participation seem unattractive to students. Rather than refuse outright, the children may not have responded positively to requests from the researchers or their teachers to access their caregiver’s current phone numbers.

### Scheduling the interview

Both the baseline and the follow-up interviews were conducted in schools, outside of regular class time, usually on Saturdays. However, because they also worked on Saturdays, several caregivers could not attend these appointments. During the follow-up, a total of eleven interviews were conducted at the university facility to enhance feasibility of caregivers’ attendance. Additionally, if they were resistant to going to school for the interview, students and their caregivers were invited to interview at the university facility instead. For those cases, a transportation reimbursement of US$ 12.00 (approximately R$40.00) was offered. Finally, if the student could not attend either the school or the university appointment, the team conducted a home-based interview. This option was used with only four subjects and ultimately had to be abandoned due to security concerns. Some subjects lived in very poor conditions, largely inaccessible by car or public transportation and in violent neighborhoods with little police surveillance. Study coordinators decided not to continue this domiciliary strategy, in order to preserve the interview team’s safety.

In the first wave, 159 interviews (88.3% of the 180 interviews) were conducted at the schools, 17 (9.4%) were conducted at the university facilities and 4 (2.3%) were home-based interviews. At the follow-up, 80 interviews (67.7% of the 118 interviews) were conducted at the schools and 38 (32.3%) were conducted at the university facilities. Home-based interviews were not made, as explained above.

### Instrument

Highly specialized professionals were required to perform the interviews and to receive the K-SADS training. However, as child and adolescent psychiatrists are scarce in Brazil, these professionals are in high demand. This project paid approximately US$ 30.00 per hour of work while clinical facilities not related to research projects usually pay US$ 60.00 for an equivalent workload. Therefore, the turnover rates of these professionals on our research team were high, which necessitated new interviewers being trained and in turn increased expenses of the project. In addition, the use of K-SADS is time-consuming both during the interview and during its manual interpretation which further increases cost of the required workforce. Each interview took 2 h (1 each for the adolescent’s and the caregiver’s interview) plus an additional 30 min for final diagnosis scores. Additionally, the interview included some intimate questions, concerning topics like alcohol and illicit substance use, domestic violence, and neighborhood conditions, which could be embarrassing to answer in a face-to-face interview and may have negatively influenced participants’ opinions about the instrument and increased information bias.

### Lack of incentive to participate

The Brazilian research legislation (Resolução CNS 196/96) is one of the toughest legislations in the world concerning the payment/reimbursement of research subjects for their time answering survey questionnaires. Any kind of incentive, financial or otherwise, is strictly forbidden [20, 21]. In Brazil, participation must be totally voluntary and incentives are seen as an impingement on free will. Therefore, attracting individuals to participate is a major concern of every field survey in the country. Teachers and school principals were asked to help the research team in involving the community in the survey, inviting caregivers to participate and explaining both the research goals and the possible implications of the findings for the community. However, most of these professionals are overloaded with multiple job responsibilities already, and thus had low motivation and little time to help with the research implementation.

In order to provide something more concrete and useful for the participants, the research team offered appointments for select students (those referred by the schools’ principals) in the CAP facility of the university. CAP facilities are scarce in the São Paulo metropolitan area and it is difficult to have a child or adolescent evaluated by a psychiatrist. During this period, a student from the public-school system not included in our sample was also evaluated every week at the CAP facility. Adolescents included in the research could only be referred to this clinical evaluation after the year of follow-up. It is important to state that this reward was offered only after data collection was completed and that only school staff was aware of this reward. This was important to prevent selection bias, as on an individual level it could be an incentive not to participate (i.e. people with active mental disorders or drug and alcohol problems might be less interested in participating, because of a desire to avoid diagnosis or referral to appointments). In addition, appointments at the drug and alcohol facility of the university were offered for school employees and for students’ parents and relatives. Throughout the two years of the study, at least twenty patients were referred to the drug and alcohol unit. Offering this kind of clinical evaluation for students and their families and for the employees of the included schools was a way to provide a sanctioned reward and to engage the community in the research project.

### Loss-to-follow-up

After one year, the research team tried to reach all 180 adolescents and their caregivers again. Ultimately, 118 adolescents and their caregivers were re-interviewed, an acceptance rate of re-interview of 65.5% (see Table 1 for detailed information). Forty-two subjects (23.3%) could not be contacted during the follow-up period due to disconnected phone numbers, even after calling the 5 telephone numbers registered during the baseline interview. Twenty participants (11.2% out of the 180 initially interviewed) refused to participate in the follow-up interview. Reasons for refusal included lack of interest in the study (70% out of the 20 who refused to participate), a feeling that the adolescent did not have a psychiatric problem anymore (20%) and dissatisfaction with the interview conducted during the previous year (10%) mainly due to time consumed.

**Table 1:** Rates of participation at baseline and at follow-up of nine different schools in the city of São Paulo, Brazil (2014–2016)

School^a^	Baseline		Re-Interviewed at follow-up		Refusal at follow-up		Not contacted at follow-up after at least five attempts	
	N	%	n	%	n	%	n	%
Neighborhood with high exposure to urban violence and low HDI^b^								
001	18	18.0	9	50.0	-	-	9	50.0
008	47	47.0	40	85.1	7	14.9	-	-
002	20	20.0	10	50.0	3	15.0	7	35.0
003	15	15.0	-	-	-	-	15	100.0
Sub-Total	100	100	59	59.0	10	10.0	31	31.0
Neighborhood with low exposure to urban violence and high HDI^b^								
004	14	17.5	6	42.8	8	57.2	-	-
005	13	16.2	9	69.2	4	30.8	-	-
000	7	8.75	5	71.4	-	-	2	28.6
009	29	36.2	7	24.1	14	48.2	8	27.7
006	17	21.1	5	29.6	6	35.2	6	35.2
Sub-Total	80	100.0	32	40.0	32	40.0	16	20.0
TOTAL	180	100.0	91	50.5	42	23.3	47	26.2

^a^Schools were numbered in order to guarantee anonymity; ^b^Human Development Index

In the end, several factors may help explain the loss- to-follow-up experienced for this specific project. Among these potential factors are difficulty in getting updated contact information for participants, a prohibitively long baseline interview with very intimate questions and a lack of concrete rewards able to attract and retain participants.

The difficulty in keeping contact information updated is likely a problem shared by other LMICs, as their governments may have trouble in gaining access to people from low SES and in maintaining a detailed database of citizens, due to lack of budget. In our project, working together with social leaders, like schools’ principals, was an important tool to reach participants in the follow-up period and may help explain why the loss rate varied among schools. Attendance rate at follow-up seemed to be a direct reflection of the effort of the school principals to motivate families to participate.

Establishing a good relationship with the school principals was crucial for study implementation. Therefore, it was important to actively work together with principals. To that end, the CFS thoroughly explained the research aims and procedures to the schools’ principals and made sure to involve them in all steps of the data collection. Each principal took part in setting the dates for the interviews, engaged in weekly updates about scheduling, provided contact information for participants and provided support staff for the research team on interview day.

Feedback about the response rate obtained and the main problems of the day were also shared with principals. After each wave of interviews, preliminary findings were presented to each school principal separately. These findings would highlight the reality of each individual school as well as compare it to other schools within the same neighborhood.

## Discussion

This study presents the difficulties faced by researchers in a school-based longitudinal pilot study conducted in a middle-income country, using a clinical interview. Achieving unbiased sampling, reaching subjects, scheduling interviews, keeping an updated register of participants’ contact information, using a thorough clinical interview without an algorithm for its scoring and maintaining a highly-trained research team are some of the difficulties described. Although all these are well-recognized issues when conducting field surveys, their impact has not been appropriately discussed in the scientific literature, especially among LMICs, where social inequalities can be a particularly influential factor.

### Sampling

Participation rates for epidemiologic studies have declined in recent years around the world [22]. Two main reasons for this may be that the refusal rate has progressively increased over years, and that it has become harder to find eligible subjects [22]. Although improvements in communication technology has made it easier to contact people in some ways, it has increased the difficulty of finding people in others. Years ago, researchers could find a list of all phone numbers from a region or city in the yellow pages. In recent years, unlisted phone numbers are increasingly common and cell phones, which allow users to change their number more easily, are more widely used [22, 23]. Despite living in a middle-income country, most of the Brazilian population has an active cell phone (84% of Brazilians have an active cell phone, including 64% of Brazilians with the lowest SES – TIC Domicílios, [15]). Therefore, the difficulty in reaching subjects cannot simply be attributed to lack of telephone access. However, in Brazil, especially among people from low SES, pre-paid cell phones are commonly used [15]. In fact, according to the National Agency of Telecommunications (ANATEL, in the Brazilian Portuguese acronym), pre-paid cell phones (a much easier way of changing numbers) were responsible for 70.2% of mobile phones in Brazil [15]. This pattern of cell phone use, whereby the most disadvantaged people are using the least reliable and least reachable form of communication, may in fact be creating an important selection bias in our study.

### Scheduling the interview

In order to optimize resources, the research team organized group efforts. Several interviews were scheduled on the same day. On this day, usually a Saturday, the whole team of interviewers went together to the schools. As there usually is no class on Saturdays, all classrooms were available to the research team. This guaranteed sufficient privacy so that the adolescent and his or her caregiver could always be interviewed in different rooms. This emphasis on privacy is critically important when sensitive issues, such as sexual behavior, substance use and mental health symptoms are investigated, as it works to reduce information bias [24].

Additionally, these group efforts reduced coordination costs, as all the work was concentrated on a single day. Large school-based visits were able to reduce costs of transportation, as many interviewers shared the same car and no transportation reimbursement was needed for participants, who usually live close to their schools. Additionally, as many interviews were scheduled for the same day, the professionals always had participants to interview and there was minimal risk of wasting resources on an unproductive day.

This model also worked to reduce the risk of violent events occurring with the research team, since the group of at least four professionals, was always together. In addition, the schools are usually located in more urban regions, even in the poorest areas, which provided an extra layer of security. Reaching the schools and performing the interviews there was much safer than conducting home-based interviews, as some participants’ houses were located in more remote, peripheral areas.

In order to improve the attendance rate, all caregivers were called a day prior to their appointment as a reminder. And then again on the day of interview, the CFS called the caregivers to state that the research team was ready and waiting for them at the school. Occasionally interviews were rescheduled to a later time on the same day if the caregiver could no longer attend the previously scheduled appointment.

Especially among low SES families, several students had younger siblings who would accompany the students or their caregivers during the interview. A member of the research staff was always in charge of taking care of these children and some kind of recreational activity, such as drawing or storytelling, was always offered in order to entertain the children.

### Instrument

Some authors state that study burden may be the single greatest obstacle to study compliance and retention [25]. And thus, interviews should be as short and as fast as possible, which encourages the use of screening instruments. Instruments should be non-intrusive, self-report tools which provide flexibility and encourage honesty. These considerations are important especially in the psychiatric field, in which very personal information is requested. However, screening instruments often do not allow for either an individualized approach or a reciprocity between the interviewer and participant, which are key characteristics of clinical interviews [26, 27].

Diagnostic instruments on the other hand enable the interviewer to improvise follow-up questions based on participants’ baseline answers [28] and allow space for participants’ individual verbal expressions [29]. In addition, diagnostic instruments generate reliable and valid diagnoses, thus strengthening the research findings and reducing the number of false negatives [19]. However, they usually depend on a qualified and experienced clinical interviewer and tend to be more time-consuming, which makes their use more expensive and less practical [30].

It is important to acknowledge that the respondent’s educational background has an impact on the time needed to complete the interview. Even short interviews may take a long time to be completed for subjects with limited educational background if they have difficulty in understanding the questions being asked of them. Although this may be more important in diagnostic interviews, it can also be true of screening instruments. Brasil & Bordin [18] reported that CBCL, originally designed to be a self-report screening instrument, should be administered by a trained interviewer among the Brazilian population, due to the low educational level of the mothers interviewed in their study [18]. This may also hold true for studies in other LMICs, where subjects tend to have fewer years of education when compared to their counterparts in high-income countries.

Many researchers in youth and family mental health do not use the more comprehensive approach in psychiatric assessment. It may be preferable to do smaller, more controlled studies that validate brief screening tools against psychiatric interviews, and then proceed with the more efficient screening tools in larger school-based surveys. The data are less nuanced and clinically precise, but at least the findings from surveys with high participation are indicative of mental distress and are more generalizable.

We hypothesize that most of our loss-to-follow-up (especially for the refusal due to dissatisfaction with the interview and at least partially for the lack of interest in the study) happened because of characteristics of the instrument used. Namely, the interview was time-consuming and some questions could have been considered invasive by the participants.

### Lack of incentive to participate

For every study, it is important that the included subjects recognize a value in their participation. This value can be abstract, like adding to scientific knowledge and providing data which may help improve social conditions, or may be concrete, such as vouchers or money. Payments can provide an incentive for participating in surveys and can increase retention rates in follow-up studies [25]. Moreover, incentives, especially monetary ones [31], increase the perception of trust, reciprocity, and appreciation on the part of the respondent [31–33].

Different reward policies for research participants are found worldwide and understanding them is important for producing comparable data and for designing multicenter studies. In a comparison with six countries from Latin America (Argentina, Chile and Mexico) and Europe (Germany, Spain, France), the Brazilian regulation system was found to be the most severe [21]. Although thorough regulation is important to ensure that human rights and research ethics are upheld, it may also act as an obstacle to recruiting participants to scientific research, especially in longitudinal studies.

As any kind of concrete reward for research participation is prohibited in Brazil, the research team had to make great efforts to engage participants, by explaining to them the scientific importance of the information provided and the possible deployments of the research. As most of the participants included in our sample were from low SES, this abstract reward may not have been enough to keep their interest in the study during the follow-up.

### Loss-to-follow-up

The use of a clinical instrument, as discussed above, may have been a barrier to follow-up attendance. Although this type of instrument provides valuable, in-depth information, it simply does not seem feasible for large samples. Therefore, the use of short and fast screening instruments is strongly recommended for large longitudinal studies. In addition, the lack of concrete rewards, as discussed above, may have influenced the follow-up attendance.

This is a major concern when conducting longitudinal studies, especially in an LMIC, as high rates of loss-to-follow-up may prevent collection of reliable, valid data.

Although loss-to-follow-up is not the best predictor of overall survey quality [31, 34], a significant loss can be a major threat to a study’s validity if it does not happen randomly across the sample [35]. This may raise concerns about study precision, nonresponse bias, and the generalization of study findings [31, 36]. Even though we recorded several phone numbers at the baseline survey and we made major efforts to engage community in the research, our loss- -to-follow-up rate was relatively high.

## Conclusion

This school-based epidemiological, longitudinal study offers some important insights about the problems faced when conducting epidemiological field work in an LMIC and provides some alternatives on how to deal with these difficulties. Working closely with community leaders, organizing group efforts to perform interviews, using a short, easy to understand instrument and providing some kind of reward for participants are some of the possible strategies to be used, not only in Brazil, but also on other LMICs.

## Footnotes

1 The Human Development Index (HDI) is a composite statistic of life expectancy, education, and per capita income indicators, which is used to rank countries into four tiers of human development. The HDI may vary from 0 (no human development) to 1 (total human development).
